# NAD^+^ pool depletion as a signal for the Rex regulon involved in *Streptococcus agalactiae* virulence

**DOI:** 10.1371/journal.ppat.1009791

**Published:** 2021-08-09

**Authors:** Thierry Franza, Annika Rogstam, Saravanamuthu Thiyagarajan, Matthew J. Sullivan, Aurelie Derré-Bobillot, Mikael C. Bauer, Kelvin G. K. Goh, Violette Da Cunha, Philippe Glaser, Derek T. Logan, Glen C. Ulett, Claes von Wachenfeldt, Philippe Gaudu

**Affiliations:** 1 Micalis Institute, INRAE, AgroParisTech, Université Paris-Saclay, Jouy-en-Josas, France; 2 Department of Biology, Lund University, Lund, Sweden; 3 School of Pharmacy and Medical Sciences, and Menzies Health Institute Queensland, Griffith University, Gold Coast, Queensland, Australia; 4 Department of Biochemistry and Structural Biology, Center for Chemistry and Chemical Engineering, Lund University, Lund, Sweden; 5 Institut Pasteur, Unité Ecologie et Evolution de la Résistance aux Antibiotiques, CNRS UMR 3525, Paris, France; 6 Institut Pasteur, Unité de Biologie des Bactéries Pathogènes à Gram Positif, Paris, France; University of Texas Medical School at Houston, UNITED STATES

## Abstract

In many Gram-positive bacteria, the redox-sensing transcriptional repressor Rex controls central carbon and energy metabolism by sensing the intra cellular balance between the reduced and oxidized forms of nicotinamide adenine dinucleotide; the NADH/NAD^+^ ratio. Here, we report high-resolution crystal structures and characterization of a Rex ortholog (Gbs1167) in the opportunistic pathogen, *Streptococcus agalactiae*, also known as group B streptococcus (GBS). We present structures of Rex bound to NAD^+^ and to a DNA operator which are the first structures of a Rex-family member from a pathogenic bacterium. The structures reveal the molecular basis of DNA binding and the conformation alterations between the free NAD^+^ complex and DNA-bound form of Rex. Transcriptomic analysis revealed that GBS Rex controls not only central metabolism, but also expression of the monocistronic *rex* gene as well as virulence gene expression. Rex enhances GBS virulence after disseminated infection in mice. Mechanistically, NAD^+^ stabilizes Rex as a repressor in the absence of NADH. However, GBS Rex is unique compared to Rex regulators previously characterized because of its sensing mechanism: we show that it primarily responds to NAD^+^ levels (or growth rate) rather than to the NADH/NAD^+^ ratio. These results indicate that Rex plays a key role in GBS pathogenicity by modulating virulence factor gene expression and carbon metabolism to harvest nutrients from the host.

## Author summary

The Rex family transcription repressors serve critical roles in control of cellular metabolism. Pathogenic microorganisms often use metabolic cues to regulate their metabolism and virulence functions. Here, we structurally and functionally characterized Rex in the facultative aerobic Gram-positive coccus *Streptococcus agalactiae*, also known as group B streptococcus (GBS). GBS is a leading cause of meningitis in newborn babies and infants. Rex interacts with both the oxidized and reduced form of the cofactor nicotinamide adenine dinucleotide (NAD). The reduced form (NADH) abrogates DNA binding whereas the oxidized form (NAD^+^) enhance binding of Rex to DNA. We show that the cellular concentration of NAD^+^ is the main effector of Rex binding to DNA. A drop in the concentration of NAD^+^ is triggered when cells do not find a specific sugar such as glucose in the environment. Transcriptomic analysis revealed that GBS Rex controls not only central metabolism, but also its own expression as well as virulence gene expression. Together our results identify GBS Rex as an important transcription regulator critical for GBS pathogenicity by modulating virulence factor gene expression and carbon metabolism to efficiently utilize nutrient resources provided by the host.

## Introduction

*Streptococcus agalactiae*, or group B streptococcus (GBS), is a Gram-positive commensal bacterium that asymptomatically colonizes the human gastrointestinal tract and the vaginal flora of 10 to 30% of healthy women. In a sub-population of humans, mainly in newborns or immunocompromised elderly individuals, GBS can become an opportunistic pathogen causing diverse pathologies from meningitis to systemic infections [1–3]. GBS can also colonize the mammary glands of ruminants, causing inflammatory disease (mastitis), and has emerged as a fish pathogen in Asia [4]. It is likely that the broad host range of GBS reflects production of numerous virulence determinants required for adhesion to and invasion of eukaryotic cells, and/or for resistance to and evasion of host immune responses [3]. For instance, an orange pigment (granadaene) protects GBS against oxidative stress that is encountered in the vacuoles of phagocytes [5]; while β-haemolysin/cytolysin (β-H/C) subverts innate immune-mediated GBS clearance [6]; extracellular nuclease (NucA) is effective at subverting neutrophil extracellular traps in the host [7]; and by degrading extracellular cyclic-di-AMP the phosphodiesterase CdnP and the ectonucleotidase NudP allow GBS to modulate immune responses [8,9]. In addition to the contributions of surface and extracellular virulence factors, GBS persistence in the host is supported by its capacity to produce energy via carbon metabolism, which promotes GBS survival and multiplication. *In vitro*, GBS can undergo fermentation or aerobic respiration. Shifts between metabolic states depend on growth conditions. Aerobic respiration requires exogenous haem and menaquinones (MK) or their common biosynthetic precursor 1,4-dihydroxy-2-naphthoic acid (DHNA) [10,11]. Indeed, endogenous synthesis pathways for haem and DHNA are absent in GBS [12]. These molecules allow activation of a respiratory chain, which is ended by a high-oxygen-affinity cytochrome *bd* terminal oxidase, CydAB. Compared to fermentation, respiratory metabolism confers many advantages to GBS, including higher biomass yield, robust senescence *in vitro* and enhanced virulence in a systemic infection model in mice, indicating its role in GBS pathogenicity [11,13,14].

In bacteria, genes implicated in central carbon and energy metabolism are typically controlled by several regulators, one of which is the transcriptional redox regulator Rex (reviewed in Ravcheev et al. 2012). Rex is postulated as a sensor of the redox poise through changes in the NADH/NAD^+^ ratio and is a repressor of transcription [15]. Sequence analysis and direct experiments show that members of the Rex family are present in most Gram-positive bacteria (Firmicutes, Streptomyces) but appear to be absent from *Mycobacterium* and from most Gram-negative bacteria [16–18]. Rex is a homodimer consisting of two domains, an N-terminal DNA recognition domain and a C-terminal Rossmann-like dinucleotide-binding and dimerization domain [15–22]. The dimerization of Rex is stabilized by the swapping of the C-terminal α-helices and the dimer binds to a consensus **R**ex **OP**erator (ROP) sequence (TTGTGAAN_4_TTCACAA) to repress expression of target genes. DNA-binding of Rex dimers with ROP-containing nucleotides is stabilized by the presence of NAD^+^, and in contrast, NADH destabilizes the complex and decreases DNA-binding affinity of N-terminal dimers through conformational change, as exemplified by *in vitro* studies of *Streptomyces*, *Staphylococcus* and *Bacillus* [15,18,23]. In complex with NADH the two N-terminal domains of Rex pack close to each other in a compact dimer. This Rex conformation prevents its binding to DNA with high affinity. Thus, it is proposed that Rex-mediated gene transcription can occur only when the NADH pool rises (or NADH/NAD^+^ ratio increase), reflecting the role of Rex as a cellular redox-state sensor. In *S*. *coelicolor* and *B*. *subtilis*, inhibition of aerobic respiration by oxygen limitation or inhibitors increases NADH levels and, as a consequence, induces expression of Rex-repressed genes, including those encoding the cytochrome *bd* oxidase (*cydAB*) and the lactate dehydrogenase (*ldh*) [15,24]. This leads to production of the CydAB oxidase, which ensures efficient oxygen usage, and lactate dehydrogenase (LDH), which recycles excess NADH. Intriguingly, Rex is also present in respiration-deficient species, such as *Clostridium difficile* (an obligate anaerobic bacterium), and *Streptococcus* species, such as *Streptococcus mutans*, *Streptococcus pneumoniae*, and *Streptococcus suis* [19,20,25]. Together, these findings indicate there are alternative mechanisms in Rex signalling pathways in bacteria that remain to be identified.

To further understand how Rex operates in Gram-positive bacteria, we investigated the structure, function and role of this regulator in the serotype III strain NEM316 of GBS isolated from a fatal case of septicaemia [12]. We characterized the Rex regulon and demonstrate its role in central and energy metabolism. In addition, we show a hitherto unexpected role for Rex in bacterial virulence. A deletion of *rex* affects expression of major virulence factors, including the β-H/C encoded by the *cyl* operon and attenuates GBS pathogenicity in a model of systemic disseminated disease. The crystal structure of GBS Rex reveals an overall organisation similar to that of other bacterial Rex proteins. Finally, we establish that GBS Rex responds to concentrations of the NAD^+^ pool in contrast to the expected NADH/NAD^+^ ratio. Together, these findings define new functions of the Rex regulon and expand our understanding of bacterial Rex regulation and role in virulence during host infection.

## Results

### The Rex regulon in GBS

**Fig 1:**
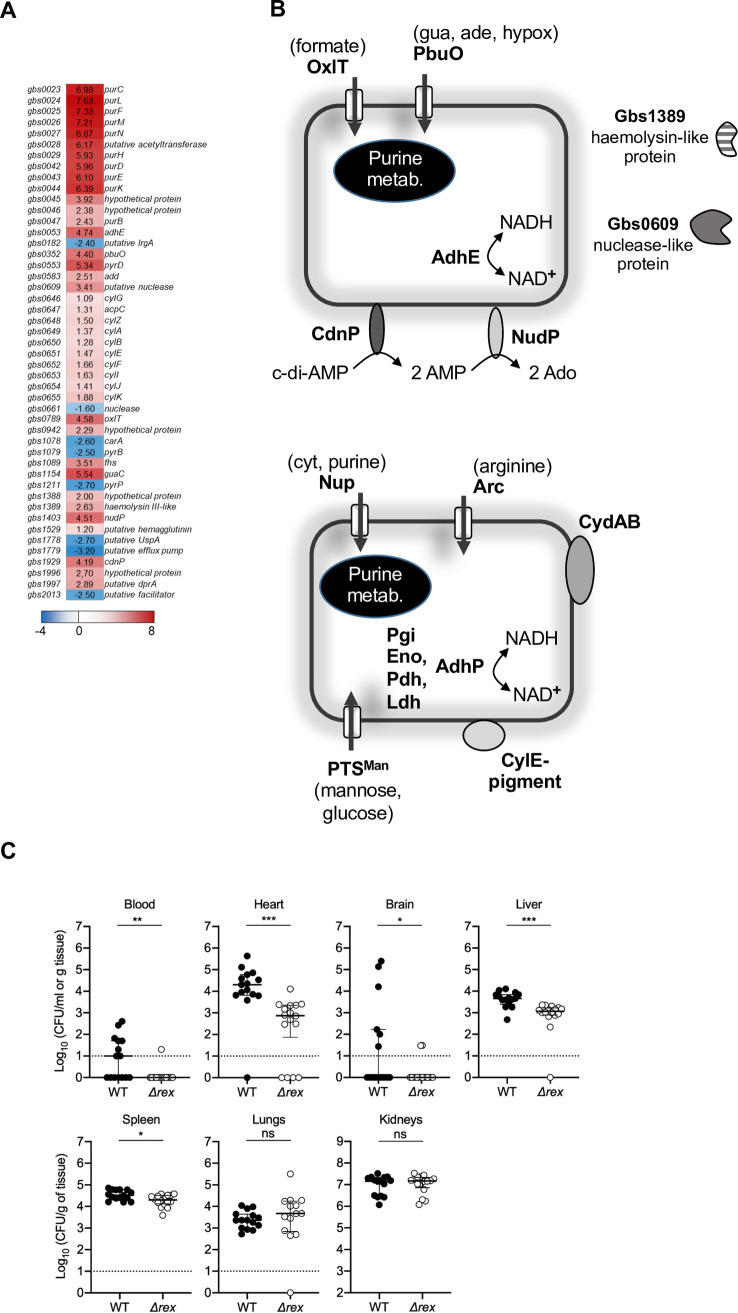
Rex regulon and its role in virulence of GBS strain NEM316. **A.** Heatmap of a selection of differentially expressed genes in a Δ*rex* mutant compared with wild type. The heatmap indicates log_2_ fold change and values that showed a >2-fold change (induced or repressed) are shown. Low to high expression is represented by a change of colour from blue to red, respectively. **B.** Rex-controlled genes involved in metabolism and virulence. The drawing is based on transcriptome analysis (S2, S3 and S4 Tables). Proteins encoded by genes, that were highly (upper panel) or modestly upregulated (lower panel) in the Δ*rex* mutant versus the WT strain, are indicated. Gua, guanine; ade, adenine; hypox, hypoxanthine; Ado, adenosine; cyt, cytosine. **C.** Effects of Rex on recovery of GBS from different organs in BALB/c mice following systemic infection; organs were collected 24-hour post-inoculation. Data are pooled from two independent experiments and show medians with interquartile ranges. *P value of <0.05; **P value of <0.01; ***P value of <0.001; Mann-Whitney U tests.

Analysis of the GBS strain NEM316 genome reveals the presence of a Rex-like protein encoded by the *gbs1167* gene [12]. This gene is located in a monocistronic locus flanked downstream by a *radC-*like gene (*gbs1168*) and upstream by a three gene operon (*gbs1164-1165-1166*) (S1 Fig). The encoded Rex protein is 74–78% identical to Rex of other Streptococci. To gain insight into the role of Rex in cellular processes we deleted the *rex* gene in GBS strain NEM316 (S1 Table) and compared gene expression (transcriptomes) between the wild type (WT) and mutant strains. Results of transcriptomic analyses carried out on cells grown to early exponential phase in a complex medium containing glucose (M17 with 1% glucose) in anaerobic or aerobic conditions are shown in S2, S3 and S4 Tables. A total of 35 genes were identified as highly differentially expressed as a result of *rex* deletion (>5-fold change, p<0.05; 28 genes up-regulated and 7 down-regulated), some of which are implicated in both carbon and purine metabolism (S2 and S3 Tables and Fig 1A and 1B). Among genes involved in carbon metabolism, we found *adhE* (>25-fold up-regulated) encoding alcohol dehydrogenase E, as an example of a typical Rex regulated gene in other bacteria [18,19,21,26], and genes encoding transporters such as OxlT (~20-fold up-regulated), involved in formate-oxalate exchange. Several genes linked to purine metabolism (*pur*) were up-regulated in the *rex* mutant under both aerobic and anaerobic conditions (Fig 1A). Expression of genes encoding purine transporters like *pbuO* or *gbs0942* (nucleoside binding lipoprotein) were also enhanced in the *Δrex* mutant (S2 and S4 Tables). In contrast, genes implicated in pyrimidine synthesis (*pyrB* and *pyrP)* exhibited reduced expression in the mutant (S3 Table). In addition, two genes encoding extracellular cell-wall-anchored enzymes (*gbs1403* and *gbs1929* encoding the ectonucleotidase NudP and the 2’,3’-cyclic-nucleotide 2’-phosphodiesterase CdnP, respectively) were highly expressed in the mutant (Fig 1A). NudP and CdnP work in concert to degrade extracellular cyclic-di-AMP (c-di-AMP) into adenosine and are used by GBS to modulate immune responses [8,27]. Finally, in the Δ*rex* mutant, we saw elevated expression of *gbs0609*, (Fig 1A) which encodes a putative extracellular DNA/RNA binding protein likely devoid of nuclease activity, due to a lack of key conserved residues in its catalytic site [7].

Our transcriptomic analyses highlighted an additional set of 132 genes the expression of which was moderately altered in the Δ*rex* mutant (≥2-5-fold change, p<0.05; 96 up- and 36 down-regulated). Some of the up-regulated genes are linked to carbon metabolism, including sugar uptake transporters (*pts*^*man/glu*^, *pts*^*man/fru*^), glycolytic enzymes (*eno*, *pgi*, *pgk*) and enzymes involved in pyruvate catabolism (*ldh*, *pdhAB*, *adhP*), respiration (*menA-ndh-cydABCD*), and arginine catabolism (*arc*) (S4 Table). Strikingly, we noted a number of virulence-associated genes that were also upregulated in the mutant, including the *cyl* operon encoding the CylE β-H/C [5,28] and a putative haemolysin III (*gbs1389*). Several genes linked to secretion, extracellular nuclease activity and stress, were down-regulated in the absence of *rex* (S4 Table).

The transcriptional changes described above were validated using reporter-gene transcriptional fusions to LacZ, with a selection of genes including *purC*, *adhE*, *adhP*, *gbs0609*, *menA*, *gbs1388*, *gbs0110* and *cdnP*, which were ~2 to ~200-fold up-regulated in the Δ*rex* background (S2B Fig). In addition, using a *rex-*promoter *lacZ* fusion, we determined that Rex is able to repress its own transcription, since we noted a >200 ± 25 fold increase in transcription from the *rex* promoter in the Δ*rex* mutant (S2B Fig). Introduction of *rex in trans* into the *Δrex* mutant restored activity levels of the *adhE* and *cdnP* promoter fusions close to those of the WT (S5 Table). In parallel, we performed phenotypic tests for analysing production of the β-H/C and extracellular nuclease. The WT and *Δrex* strains were grown on sheep blood agar or Methyl green-DNA indicator agar plates (S3A Fig). Deletion of *rex* led to an increased production of the β-H/C, consistent with the observed induction of expression of the *cyl* operon (S4 Table). Complementation with *rex* restored pigment production in the mutant to levels approaching the WT (S3A Fig). Global nuclease activity, as detected using Methyl green agar plates was not affected by the *rex* deletion (S3A Fig) in agreement with the absence of nuclease activity in Gbs0609 [7]). Introduction of oxygen into the culture had a modest effect on the expression of Rex target genes under the tested conditions (S2, S3 and S4 Tables). Taken together, these data show that Rex controls the expression of a suite of metabolic and key virulence genes in GBS strain NEM316.

### Rex contributes to virulence

To explore the role of Rex in GBS pathogenicity, we first monitored the survival of mice for ten days after intravenous injection of the bacteria into the bloodstream. Challenge with the WT strain led to euthanasia of > 80% of the population within two days whereas the mutant was attenuated in pathogenicity and the comparative rate of euthanasia was significantly less at 50% (S3B Fig, P value of < 0.0001). We then investigated the effects of Rex on GBS colonization capacity in various organs at 24 hours after bloodstream infection in mice. The Δ*rex* mutant was attenuated in the bloodstream, heart, brain, liver and spleen, with significantly fewer Δ*rex* mutant bacteria recovered from these tissues compared to the WT in BALB/c mice (Fig 1C). In the case of heart, the numbers of recovered Δ*rex* mutant cells were almost 100-fold lower than the WT. Similar attenuation of the Δ*rex* mutant was observed in several organs, including the heart, liver and kidneys in C57BL/6 mice (S3C Fig). In contrast, we detected no significant differences in bacterial counts between the Δ*rex* mutant and WT recovered from the lungs of mice (Fig 1C). Collectively, these results demonstrate that Rex enhances GBS virulence during systemic infection.

### Nucleotide binding to Rex

For *in vitro* studies, the GBS Rex protein was produced in *E*. *coli* and purified to homogeneity. Isothermal titration calorimetry (ITC) was used to obtain a measure of the binding affinities of different nucleotides for Rex. A solution of 10 μM Rex was titrated with nucleotide solutions (NADH, NAD^+^, NADPH, and ATP). Examples of raw data and fitted isotherms are shown in S4 Fig. It is possible to fit the data for all nucleotides with a one-site model, and association constants and binding enthalpies are given as weighted averages in Table 1. The stoichiometry of the binding reaction with NADH was close to 1 (0.90 ± 0.08). Due to the low affinity for the binding of NAD^+^, NADPH, and ATP the stoichiometry of the reaction could not be unambiguously determined from the experimental data and it was assumed to be 1:1. Among the different nucleotides tested only NADH binds with high affinity (K_a_ = 2.8 ± 0.5 × 10^6^ M^−1^). On the other hand, NAD^+^ showed a 215-fold lower affinity to Rex (K_a_ = 0.013 ± 0.003 × 10^6^ M^−1^) but it was increased in the presence of DNA (K_a_ = 0.066 ± 0.4 × 10^6^ M^−1^).

**Table 1:** Nucleotide binding to Rex as measured by isothermal titration calorimetry.

Ligand	K_a_ (M^-1^)^a^ x 10^−6^	ΔH(kcal/mol)^a^
NADH	2.8 ±0.5	-24±3
NAD^+^	0.013 ±0.003	-21.6±0.5
NAD^+^^b^	0.066 ±0.4	-6.7±2.5
NADPH	0.007±0.002	-13±1
ATP	0.004 ±0.0006	-13±5

^a^ Binding affinities and binding enthalpies are given as weighted averages with one standard deviation.

^b^ Titration into a solution with Rex bound to DNA.

### Rex binding to DNA

**Fig 2:**
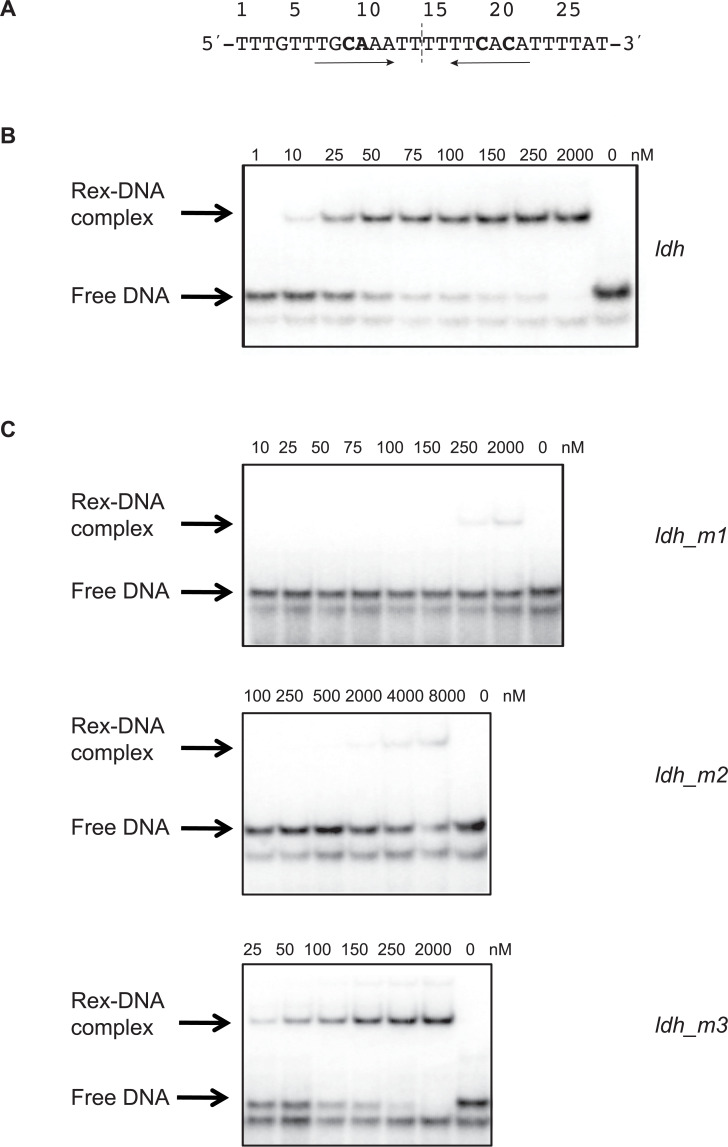
*In vitro* binding of Rex to an operator sequence. EMSA assays of ^32^P-labeled double stranded DNA fragments containing the predicted *ldh* Rex operator. The concentration of Rex in the binding reaction is indicated above each lane. **A**. Sequence of the *ldh* operator fragment. The axis of symmetry is indicated by the dotted line and nucleotides that were exchanged are shown in bold. **B**. Complex formation with native *ldh* operator fragment. **C**. Complex formation with mutated *ldh* operator fragment: *ldh_m1* (C19→T), *ldh_m2* (C19→T, C21→T), *ldh_m3* (C9→T, A10→G).

*B*. *subtilis*, *S*. *aureus* and *S*. *coelicolor* Rex binds to an 18 bp inverted repeat Rex operator (ROP) sequence [15,18,23]. In both *B*. *subtilis* and *S*. *aureus*, the gene encoding L-lactate dehydrogenase (*ldh*) is regulated by Rex. A 28 bp DNA fragment containing a putative ROP site upstream of the GBS *ldh* gene (Ldh, *gbs0947*) (Fig 2A) was incubated with purified *S*. *agalactiae* Rex, and the bound complex was separated by native PAGE in an electrophoretic mobility shift assay (EMSA) (Fig 2B). As controls, we tested binding to three variants of the putative *ldh* ROP site containing transitional mutations; *ldh_m1* (C19→T), *ldh_m2* (C19→T, C21→T), and *ldh_m3* (C9→T, A10→G) (Fig 2C). In the first two variants, nucleotides previously shown to be important for Rex binding in *S*. *aureus* were exchanged. In the third variant the introduced changes produce a ROP binding site with two almost identical half sites resembling the *S*. *aureus* ROP consensus sequence (TGTGAN_6_TCACA). The EMSA analysis showed that Rex bound very weakly to *ldh_m1* (C19→T) and not to *ldh_m2* (C19→T, C21→T). Mutations in *ldh_m3* (C9→T, A10→G) had no effect in the binding compared to the original *ldh* ROP site (Fig 2C). The ITC and EMSA analysis indicate that Rex binds to ROP-DNA with a dissociation constant of approximately 30 nM. The stoichiometry of the binding reaction with ROP-DNA was close to 2 (2.2 ± 0.2), indicating that one Rex dimer binds per DNA molecule. It has previously been shown that *B*. *subtilis* Rex is allosterically activated for DNA binding by NAD^+^ [23]. It was therefore of interest to analyse if NAD^+^-bound Rex influenced DNA binding in GBS. We first prepared a Rex solution free of any NAD(H) (apo Rex) as some Rex can contain NAD(H) [22]. By using ITC, titration of NAD^+^ into a mixture of Rex and DNA demonstrated that NAD^+^ increases Rex affinity for DNA by approximately 35% compared to that determined with apo Rex, indicating a positive allosteric effect in binding of DNA and NAD^+^.

**Fig 3:**
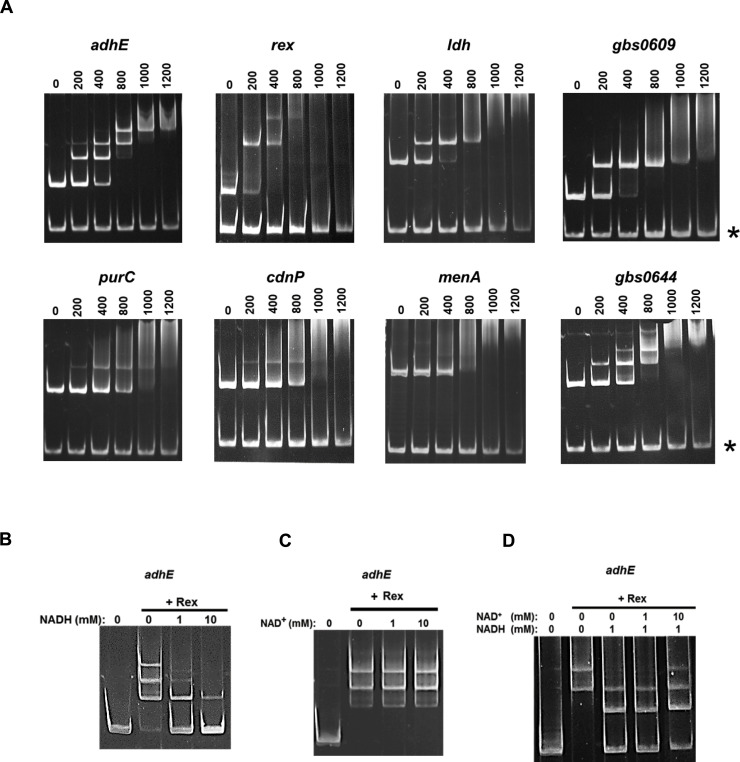
Rex directly binds to the promoters of a selection of upregulated genes. **A**. EMSAs with purified Rex protein and promoter DNA fragments from the *adhE*, *rex*, *ldh*, *gbs0609*, *purC*, *cdnP*, *menA* and *gbs0644 (cyl)* genes. DNA fragments (30 nM) were incubated with the indicated concentrations (in nM) of Rex protein for 20 min at 30°C. Then, the protein-DNA complexes were resolved by electrophoresis on TBE polyacrylamide gels. As a negative control, an internal fragment of 170 bp from the *gbs0455* gene, lacking *rex* binding site, was used as a DNA competitor (indicated by an asterisk). **B.** EMSAs were performed with the *adhE* promoter fragments (30 nM), Rex protein (600 nM), and the indicated concentrations of NADH. No Rex protein was added to the first lane. **C.** EMSAs were performed with the *adhE* promoter, as in A, but with different concentrations of NAD^+^. **D.** EMSAs were performed with the *adhE* promoter, as in B, but with different NADH and NAD^+^ concentrations.

Next, we performed additional DNA-binding assays with PCR-amplified DNA fragments from the promoter regions of selected genes (Fig 3A) containing one or more putative ROP sequences situated close to the -35 and -10 promoter elements (S2A Fig). An internal fragment of the *gbs0455* gene that does not contain a sequence close to the ROP consensus was used as a DNA binding competitor. In EMSA, the promoter regions of the *adhE*, *rex*, *ldh*, and *gbs0609* genes displayed a dose-dependent mobility shift to higher molecular mass (Fig 3A), indicating the formation of specific high affinity Rex–promoter DNA complexes. The *adhE*, *rex* and *gbs0644* promoter regions display several mobility shifts which can be caused by Rex oligomerization around the operator or by the presence of more than one Rex operator as it occurs for the *adhE* promoter (S2A Fig). A less prominent but specific mobility shift was observed for the promoter regions of *purC* and *cdnP* indicating that Rex binds with a lower affinity to these sequences. No mobility shift was observed for the *menA* promoter region suggesting that the *menA-ndh-cydABCD* operon is not directly regulated by Rex. These results are consistent with the transcriptional observations shown in S2B Fig. Using the ROP sequences from selected genes (S2A Fig), a 21-nucleotide consensus sequence 5’-TTGTWNNNNNNTTCACAWNNT-3’ was generated using the MEME algorithm [29] with C nucleotides at positions 14 and 16 being important for Rex binding (Fig 2C). We used this consensus sequence to predict the Rex regulon in *S*. *agalactiae* (S6 Table).

### Effects of NAD(H) on Rex binding to DNA

The ITC data presented above showed that Rex binds the ROP sequence with an affinity that is only modestly increased by the presence of NAD^+^, while the opposite is expected with NADH based on prior studies of Rex from other bacteria [15,18,23]. To test whether NADH affects the interaction between Rex and its cognate operators, EMSAs were performed with *adhE* DNA fragment and Rex preincubated with increasing concentrations of NADH (Fig 3B). Formation of DNA-protein complexes was partially inhibited by 1 mM NADH, and 10 mM NADH almost completely abolished binding activity of Rex to the promoter sequence. In contrast, there was no clear effect of NAD^+^ on Rex DNA-binding activity in the absence of NADH in the EMSA assay (Fig 3C), probably because a fraction of the purified Rex preparation used for the EMSA assay already contained NAD^+^. Indeed, reduction of the Rex protein preparation with dithionite yielded an absorbance peak at 340 nm, indicating formation of NADH (derived from reduction of Rex-bound NAD^+^) (S4G Fig). Increasing concentrations of NAD^+^ were able to partially restore the binding of Rex to the *adhE* DNA fragment in the presence of 1 mM NADH (Fig 3D), suggesting that a 10-fold excess of NAD^+^ can partially displace NADH in the Rex protein. Together, these results suggest that native Rex binds NAD^+^ and directly represses target genes by binding at operator specific sites *in vivo*.

### Crystal structures of Rex with NAD^+^ and its interaction with ROP DNA

**Fig 4:**
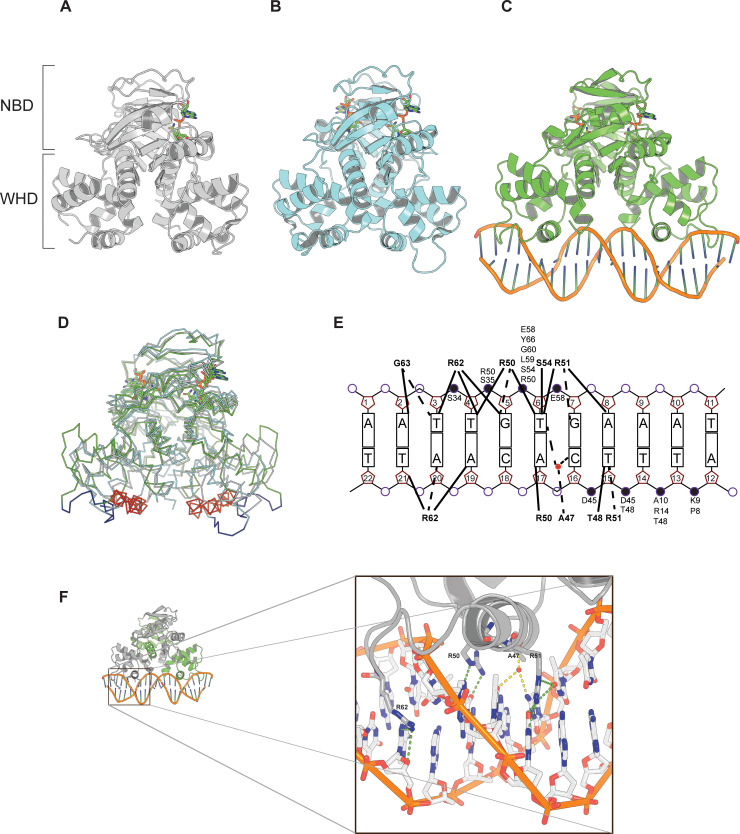
Structures of Rex alone and in complex with Rex ROP DNA. Cartoon representations of Rex in complex with NAD^+^ (**A** and **B**) and NAD^+^ and DNA (**C**). The nucleotide binding domain (NBD) that binds NAD^+^ and NADH and the winged helix domain (WHD) that interacts with DNA are indicated. **D**. Superposition of the three structures. The wing of the WHD is coloured blue and the recognition helix (α-helix 3) that makes sequence-specific DNA contacts is coloured red. **E**. Schematic representation of protein–DNA interactions in the Rex-ROP complex. Due to the symmetric nature of the complex only half of the 22 bp DNA is shown. Hydrogen bonds are shown as dotted lines and van der Waals contacts as solid lines. Residues that make contacts with the phosphate moieties are indicated. **F**. Overall structure of the Rex-DNA complex with one Rex subunit coloured grey and the other green. Close up of the Rex-DNA interactions (corresponding to the boxed region). For clarity only the wing and the recognition helix of Rex is shown. Residues that form hydrogen bonds (dotted green lines) to the bases of DNA are labelled and shown as sticks. Water-bridged hydrogen bonds between Ala47 and bases of DNA are shown as dotted yellow lines.

We determined three crystal structures of Rex: in complex with one NAD^+^ per dimer at 2.4 Å resolution (NAD1, PDB 3KEQ), in complex with two NAD^+^ per dimer at 1.5 Å resolution (NAD2, PDB 3KEO) and DNA-bound at 2.4 Å resolution (PDB 3KET) (Fig 4A, 4B and 4C). In all three structures Rex is comprised of a C-terminal domain-swapped dimer composed of an N-terminal DNA binding domain linked to a NAD-binding Rossmann-like domain. This arrangement is shared between all determined Rex structures [15–22]. The GBS Rex structures are described in more detail below and serve as the basis for gaining a more detailed insight into the conformational changes upon nucleotide and DNA binding. The refinement statistics and model quality parameters are listed in Table 2. The Rex-NAD^+^ complex crystalized in two different space groups, P2_1_ and P2_1_2_1_2_1_. The higher resolution P2_1_ form contained two NAD^+^ molecules per Rex dimer whereas the P2_1_2_1_2_1_ form only contained one NAD^+^ molecule per dimer. The structure of Rex in complex with a palindromic 22 bp dsDNA fragment containing the ROP sequence (5’-AA**TTGT**G**AA**ATAT**TTCACAA**TT-3’, the conserved ROP consensus sequence from different bacterial species including GBS is indicated in bold) was obtained by co-crystallization and belonged to space group I222. The asymmetric unit contains a single Rex monomer bound to the corresponding half site of the ROP DNA which is predominantly in B-form, with the Rex dimer and complete ROP site related by crystal symmetry. The superimposition of the Rex-NAD^+^ complex and the DNA bound conformers revealed root-mean-square deviations (RMSD) ranging from 3.1 Å (for NAD1) to 4.0 Å (for NAD2). These changes are confined primarily to rearrangements of the N-terminal DNA binding domain (Fig 4D). In order to bind to the ROP the DNA binding domain of one of the subunits of the Rex-NAD^+^ complex must rotate 26.2^o^. The rotation leads to an increased distance between the recognition helices (α3) from NAD1 (18 Å), NAD2 (15 Å to 34 Å).

**Table 2:** X-ray data and refinement parameters.

Measurement	Rex–NAD2 (3KEO)	Rex–NAD1 (3KEQ)	Rex–DNA (3KET)
Space Group	P2_1_	P2_1_2_1_2_1_	I222
Cell Dimension	a = 46.27, b = 92.41, c = 47.19 Å; β = 91.26°	a = 57.67, b = 71.47, c = 113.88 Å	a = 48.27, b = 107.03, c = 153.95 Å
Beamline	I911-2	I911-2	I911-2
Wavelength (Å)	1.03	1.03	1.03
Resolution (Å)	1.50 (1.54–1.50)	2.40 (2.46–2.40)	2.40 (2.46–2.40)
No of unique reflections	62064 (4374)	18880 (1369)	16157 (1159)
Completeness (%)	98.0 (93.3)	99.4 (100)	99.6 (97.9)
Redundancy	3.9 (3.5)	6.0 (6.1)	4.7
*I*/σ	8.4 (2.3)	27.3 (3.7)	10.2 (3.0)
R_sym_ (I) (%)	8.4 (52.8)	4.2 (52.3)	10.6 (46.0)
**Refinement**			
No. of residues in asymmetric unit	413	400	205 + 21*
No. of NAD^+^	2	1	1
No. of water molecules	544	59	119
R_model_ / R_free_ (F) (%)	17.5 / 23.4	22.3 / 29.2	19.5 / 24.7
**RMS deviations from ideal geometry**			
bonds (Å)	0.025	0.017	0.019
angles (°)	0.002	0.001	0.002

Statistics for the highest-resolution shell are shown in parentheses

* Nucleic acid residues

### Molecular interactions between Rex and ROP DNA

The DNA-binding domain of Rex is composed of four α-helices and two β-strands that form a winged helix-turn-helix motif (Fig 4C and 4F). The combined DNA-binding region of the Rex homodimer presents a striking electropositive surface that complements the bound DNA (S5 Fig). Each Rex monomer makes a large number of phosphate and base contacts, allowing for high affinity DNA binding (Fig 4E and 4F). The DNA-binding specificity of Rex is mediated by residues from both its wing and its helix-turn-helix regions. The wing comprises the connecting loop (residues 56–65) between helix α3 (residues 45–55) and strand β2 and packs along the side of the DNA, spanning the phosphate backbone of both strands on either side of the minor groove, involving both nonspecific contacts with the phosphate backbone, as well as specific hydrogen bond interactions between side chain of Arg_62_ (NH1) from the wing and N3 of adenine 3’ in the minor groove (Fig 4F). The recognition helices (α3) of the winged helix-turn-helix motifs of the Rex dimer bind two consecutive major grooves of the ROP. Base-specific interactions involve Arg50 and Arg51 of helix α3. Specifically, nitrogen atoms (NH_2_) of Arg50 and Arg51 make hydrogen bonds to guanine 5 O6 and to guanine 7 O6, respectively. Ala47 makes water-mediated contacts with the bases (T6 and C16) of the major groove. In addition, residues of helices α1 (Pro8, Lys9, Ala10 and Arg14), α2 (Ser34 and Ser35), α3 (Asp45, Thr48, Arg50, Arg51, Ser54) and strand β2 (Tyr66) of the wing (residues 58 to 65) are involved in non-specific contacts with the phosphate backbone (Fig 4E).

### Growth rate as an effect of carbon source is a signal for rex gene regulation

**Fig 5:**
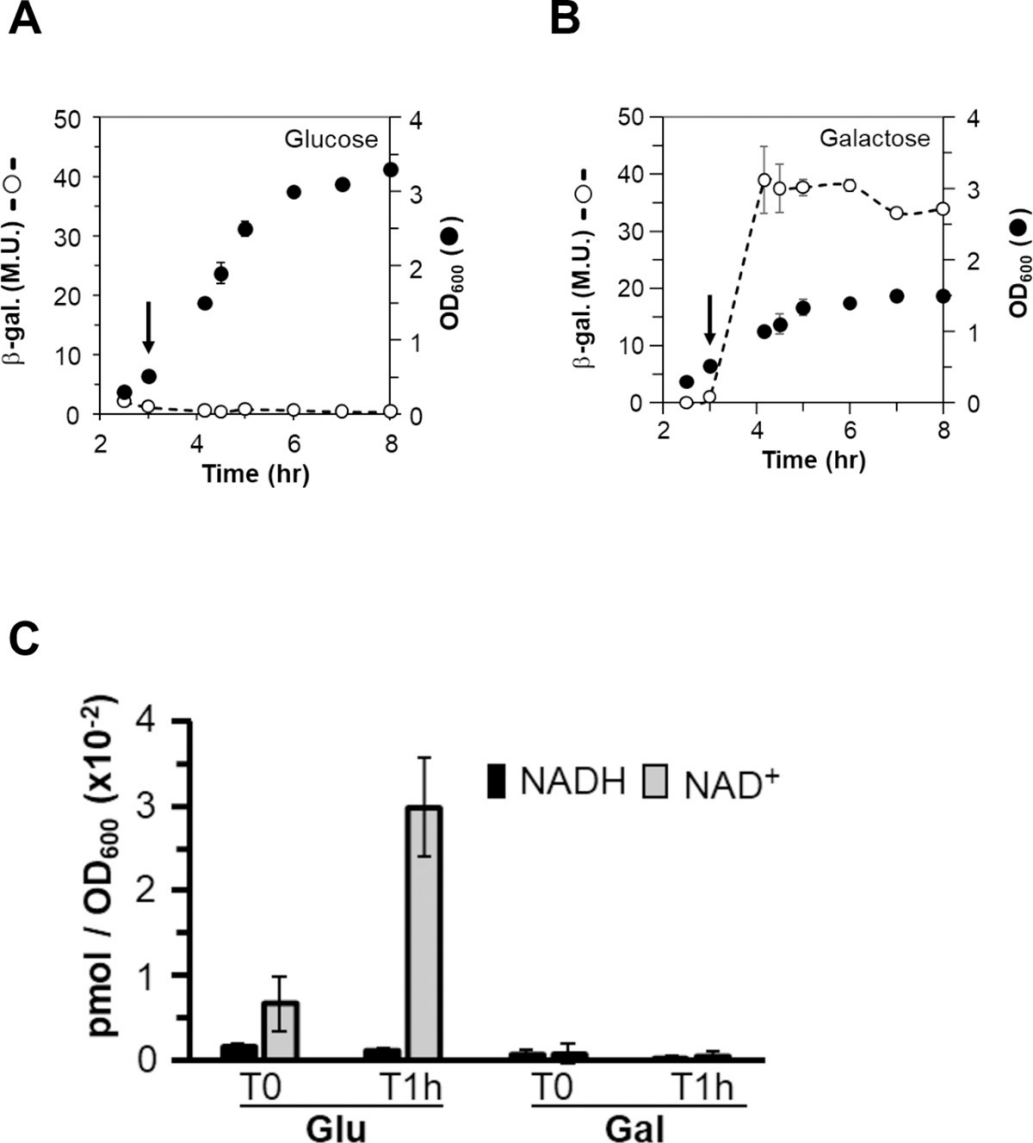
Galactose increases *rex* expression and decreases the NAD^+^ pool. WT cells containing a *rex* promoter-*lac* fusion on plasmid (Prex-*lac* plasmid) were grown in M17 supplemented with 0.5%glucose (**A**) or 0.5% galactose (**B**). When static cultures reached OD_600_ = 0.5 (indicated by an arrow), they were shifted to aerated condition and grown up to stationary phase. β-galactosidase activities (β-gal.) are expressed in Miller units (M. U.). Data are representative of at least three experiments. Mean values and standard deviations are shown. (**C**) NAD(H) pool determination. Cells were grown in the presence of glucose or galactose (0.5%) and collected at OD_600_ = 0.5 (T0) and 1 hour after the static/aerobiosis shift (T1h). Data are the mean of three independent experiments and standard deviations are shown.

It has been proposed that Rex regulators respond to the intracellular NADH/NAD^+^ ratio in bacteria. Therefore, we investigated whether this mechanism was also conserved in GBS by comparing activities of the *rex* promoter via a transcriptional fusion (plasmid P*rex-lacZ*) in the WT strain and a *nox2* mutant. This mutant harbour a disruption in the gene encoding a cytosolic NADH oxidase (*gbs0946*) and accumulates NADH [13]. In the WT strain, the *rex* promoter-*lacZ* fusion had very low activity in M17 glucose medium. In contrast, in the *nox2* mutant, a >10-fold increase of LacZ activity was observed (S6A Fig). We used the reporter strain (plasmid P*rex*-lacZ in WT) to screen for physiological conditions effective at inducing the Rex regulon. Several tested conditions failed to derepress *rex* expression, including BHI agar plates supplemented with haem (5 μM) or containing (D)MK (1 mM), DHNA (1 mM), hydrogen peroxide (H_2_O_2_, 10 mM), NaCl (1 M), lactic and acetic acids (20 mM each), antibiotics (tetracycline, chloramphenicol, vancomycin, 5 μg.ml^-1^ each), nitric oxide donors (1 mM), and the bacteriocin nisin (50 ng.ml^-1^), respectively. However, we noted that the Δ*rex* mutant displayed a growth defect under respiration versus fermentation in M17 with 1% glucose, and a growth delay in Todd-Hewitt broth (THB containing only 0.2% glucose) in comparison to the WT (S6B, S6C, and S6D Fig). To establish if these observations were linked to carbon source usage, the effect of different carbon sources on Rex regulation was tested. When grown on M17 plates with galactose or lactose, colonies of WT GBS containing the *rex* promoter-*lacZ* fusion reporter appeared blue, indicating de-repression of the Rex, while white colonies were observed when the same strain was grown on plates with glucose, maltose or mannose. In liquid culture, growth of the same strain in M17 medium with glucose proceeded with a rapid growth rate, high final biomass yield and low LacZ activity from the *rex-*reporter (Fig 5A). In contrast, in M17 medium with galactose the growth rate of WT GBS with the *rex-lacZ* fusion was low but showed high LacZ activity (Fig 5B). The addition of glucose to the galactose medium decreased LacZ activity to a similar level as observed with glucose only media, indicating that the presence of glucose restores repression of the *rex* regulon (S6E Fig). In a Δ*rex* mutant background, LacZ activity was high regardless of energy source (S6F Fig).

### Decreased cytosolic NAD^+^ concentration is correlated with derepression of the Rex regulon

To determine whether galactose-glucose effect correlated with a change of the intracellular NADH/NAD^+^ ratio, we measured the concentration of NADH and NAD^+^ in WT GBS cells growing in the presence of glucose and galactose (Fig 5C). Samples were collected immediately prior to and 1 hour after aeration, which caused maximal *rex* gene induction (Fig 5B). We extracted NAD^+^ and NADH molecules from acid and basic treatments, respectively. Before and after aeration, the NAD^+^ and NADH amounts were low in cells growing in the presence of galactose with a ratio of approximately 1 (Fig 5C). In contrast, when glucose was present a low amount of NADH was detected but remained stable during 1 hour of aerobiosis. However, the concentration of NAD^+^ increased significantly after the shift, leading to a decrease in the NADH/NAD^+^ ratio from 1/4 to 1/30. For samples from cells grown in the presence of galactose, we also tried to break the cells after an acidic hydrolysis step with beads and 10-fold more culture volume, but this method did not change the results. If we estimate the volume of a spherical cell to 0.07 μm^3^ (radius 0.25 μm) and 1 OD_600_ = 4 x10^8^ cells, the total concentrations were around 43 mM and 1.6 mM for NAD^+^ and NADH, respectively at the 1h-aeration point. These results suggest that, in the presence of glucose, the increased concentration of NAD^+^ stabilizes Rex at the ROP sequences in agreement with the ITC data (Table 1) and Fig 3D (leading to repression), whilst the NADH pool remained low.

To summarize, these results show that with glucose as a carbon source, both the growth rate and the NAD^+^ pool are high, which leads to NAD^+^ binding to Rex, and repression of Rex controlled genes (S7A Fig). With galactose as carbon source, the growth rate is lower and the NAD^+^ and NADH pools are equally low, leading to increased availability for NADH binding to Rex because of its higher affinity, and thus de-repression of Rex-controlled genes (S7B Fig).

## Discussion

During colonization and persistence in a host, the capacity of a pathogen to manage and modify central and energetic metabolism (fermentation, respiration) through regulatory networks is crucial for its survival. Previously, we demonstrated that GBS can grow using fermentation or conditional respiration depending on environmental growth conditions [11]. Respiration requires external haem and menaquinones or its precursor (DHNA). A respiration-deficient mutant was found to be attenuated in a murine model [11,13], and we therefore proposed that respiration may be the energy mode preferentially used by GBS during infection. Rex proteins are global transcriptional regulators that play a pivotal role in the regulation of energy metabolism [26], which led us to characterize the Rex protein (Gbs1167), its regulon and effects on virulence in the GBS strain NEM316, representative of the most virulent serotype group (III).

Our transcriptome analysis showed that Rex represses genes linked to central metabolism, including those encoding enzymes involved in glycolysis (enolase, Pgi glucose-6-phosphate isomerase) and pyruvate catabolism (Ldh, CydAB, AdhP, AdhE). These enzymes likely contribute to maintenance of the NADH/NAD^+^ redox balance by oxidizing free reductant excess present in the form of NADH. Of these, our data reveal *adhE* as a major Rex target that is markedly upregulated in the absence of Rex. In addition, genes associated with purine synthesis/metabolism, involved in degradation of extracellular cyclic-di-AMP (*cdnP* and *nudP*), and those encoding extracellular virulence factors such as the β-H/C and Haemolysin-like protein (HlyIII) were highly expressed in the Δ*rex* mutant. EMSA analysis showed that for some of these, Rex directly effects their expression (Figs 2 and 3A). This regulon should allow GBS to manage energy resources and likely growth during infection.

In host tissues and blood, concentrations of reduced carbon sources such as glucose are relatively low at around 0.1 mM (0.002% in blood) suggesting that finding this sugar becomes an absolute priority for GBS to grow. *In vitro*, GBS can efficiently utilize alternative glucose epimers (mannose) and maltose (1-α-D-glucopyranosyl-4-α-D-glucopyranose), which may be present in a host as alternative carbon sources that GBS can exploit. However, only monosaccharides are absorbed by the human host from the gut, which strengthens the view that glucose is a relevant carbon source used by GBS. Thus, it would appear that the Rex regulon of GBS can modulate its expression according to the energy source availability in the host, and this is detected by directly sensing NAD^+^ as an indicator of metabolic state to repress target genes. Rex also controls expression of genes involved in pathogenicity, including the *cyl* operon, *gbs1389* encoding a haemolysin-like protein, and the *nudP* and *cdnP* genes. β-H/C (and the putative haemolysin III protein) likely effects the disruption of host cellular membranes to release nutrients such as amino acids, carbohydrates, and vitamins that may be used by GBS for growth. GBS can produce and excrete cyclic-di-AMP for reasons that are not yet understood, a portion of this pool being degraded by NudP and CdnP to adenosine. Cyclic-di-AMP is a signal molecule that can be detected by the host to induce an immune response [8]. However, another explanation for GBS-mediated production and degradation of cyclic-di-AMP is to release extracellular adenosine. Bajgar and Dolezal [30] recently demonstrated that extracellular adenosine present in the bloodstream can act as a signal for increasing glucose availability at the expense of glycogen, a storage energy source, and modulates host-pathogen interactions in *Drosophila*.

The attenuation of the Δ*rex* mutant in our study was due to a reduced capacity for survival in various organs, including blood, heart and brain. These observations may be explained by the fact that de-repression of the Rex-controlled genes likely leads to extensive metabolic remodelling in the mutant. A functional Rex regulator may thus be important for bacteria to adapt to changes in their environment during host colonization and persistence. We detected significant roles for *rex* in promoting virulence of GBS in several tissues, but such a role was not observed in lungs. Oxygen partial pressure, as a reflection of the balance between oxygen delivery and consumption, differs markedly between tissues as do glucose availability, which is significantly lower in the lungs compared to other organs or in the blood [31,32]. It is worth to note that the Rex response is exacerbated by the presence of oxygen (Fig 5B), which is available at distinct levels in different host tissues. It is thus plausible that such differences might influence the role of Rex in supporting pathogenicity in different tissues. Future studies comparing the activity of Rex in different tissue compartments would be of interest as well as consideration of the impact of Rex in hyperglycaemic disease states. Interestingly, *S*. *aureus*, an opportunistic pathogen, produces a surface protein that binds insulin, which is predicted to increase glucose availability [33]. Disturbance of glucose homeostasis may be a strategy exploited by various pathogenic bacteria to ensure their growth and dissemination in host.

The structural basis of the GBS Rex regulation has been characterized in this study. GBS Rex is similar to Rex from other Streptococci (S1 Fig). In each monomer a DNA binding domain is linked to a C-terminal NAD^+^-binding domain. The DNA-binding form and the NAD^+^ bound form of GBS Rex show near-identical structure to Rex family members from mesophilic (*B*. *subtilis*) and thermophilic bacteria [23,34–37]. The conformational changes that occur in Rex upon binding of NADH can be described as a calliper-like movement, where the recognition domains are bent inwards, about 15° each, closing the angle to the dimer interface by 30° to create a compact, closed form, incapable of binding DNA. Comparisons of the three GBS Rex structures reveal significant inter-domain movement between the nucleotide binding domain and the DNA binding domain. Similar to a proposed model for *B*. *subtilis* Rex [23], binding NAD^+^ does not lock the protein in the DNA binding conformation, but rather appears to act to increase the rigidity of Rex, thereby limiting the conformational flexibility of the protein and stabilizing the interaction with DNA. The allosteric effect of DNA on NAD^+^ binding observed in this study of GBS Rex supports this idea.

Several lines of evidence support the view that elevation of the cellular NADH pool affects the NADH/NAD^+^ ratio, leading to de-repression of the Rex regulon. Low oxygen tension, inhibition of the respiratory chain, and mutation in respiratory NADH dehydrogenase [15,18,38] elevated the NADH pool concomitantly with induction of Rex-regulated gene expression. However, this signalling pathway is often linked to a respiratory activity and cannot be extrapolated to respiration-deficient bacterial species. Our studies show that GBS *rex* is expressed at very low levels in medium supplemented with glucose, likely due to high turnover of NADH (produced from glycolysis) into NAD^+^ as a product. Under these conditions with a high growth rate, the rate of flux from NADH to NAD^+^ likely exceeds the recycling of NAD^+^ back to NADH (by NADH dehydrogenases: lactate dehydrogenase, respiratory chain, NADH oxidase 2) and leads to Rex binding NAD^+^ preferentially over NADH. Galactose metabolism requires the Leloir pathway and feeds into glycolysis via glucose-1-phosphate. However, GBS does not possess the necessary enzymes for this pathway (see KEGG pathway san00052 for *S*. *agalactiae* NEM316, which lacks *galK*, *galT* and *galM*), so galactose likely does not contribute to the NAD(H) pool. As shown in Fig 5C, the NAD(H) concentration is at the limit of detection under our conditions. In this context NAD^+^ cannot bind to Rex, which releases the pocket for NADH. As the affinity of Rex for NADH is ~ 42-fold higher than for NAD^+^ in the presence of the ROP sequence (Table 1), NADH should bind Rex even at trace levels. When GBS encounters glucose again, Rex present in excess (due to the negative feedback regulation) has to switch off rapidly the regulon concomitantly with NAD^+^ pool restoration. Our regulation model explain the presence of Rex in respiration-negative species like Group A Streptococci. These organisms display similarities to GBS such as a growth defect when galactose or lactose are used as energy sources [39]. In conclusion, this work highlights the close link between bacterial pathogenicity and the availability of an energy source in the host organism.

## Methods

### Ethics statement

Animal experiments were approved by Griffith University Animal Ethics Committee (approval no. MSC/01/15/AEC) and by INRAE institutional guidelines of good animal practice (Jouy en Josas, France) and approached by the direction des Services Vétérinaires (accreditation number 78–63).

### Growth conditions

GBS strains (S1 Table) were routinely grown in Brain Heart Infusion (BHI) broth overnight under static conditions in a 5% CO_2_/95% air incubator at 37°C to prepare pre-cultures. Growth experiments were carried out using BHI, Todd Hewitt Broth (THB) or M17 broth with riboflavin 5 μM (M17rib) and supplemented with different sugars (0.5–1% glucose and/or 0.5% galactose, or 0.5% lactose). Cultures were usually inoculated at OD_600_ = 0.025 in M17rib and incubated under static conditions. For aeration conditions, M17 broth was supplemented with 50 μg ml^-1^ of haemoglobin (Sigma). Culture volume was 1/10 of flask volume, with agitation at 125–200 rpm. For respiration, aerated cultures were supplemented with 30 μM of menaquinone (MK-4 Sigma). *Escherichia coli* strain TG1 was used as host strain for cloning. When required, antibiotics were used as follows: for GBS, erythromycin (Ery) 1 μg.ml-1, chloramphenicol (Cm) 5 μg.ml-1, and for *E*. *coli*, Ery 150 μg.ml-1, ampicillin (Amp) 100 μg.ml-1. For electroporation, GBS strains were grown in BHI.

### Microarray experiments

*The S*. *agalactiae rex mutant (Δgbs1167) and WT strain were* cultivated in M17rib with 1% glucose at 37°C under anaerobiosis and aerobiosis (shaking 200 rpm). The total RNA was extracted as previously described [28] at exponential growth phase (OD ~ 0.3). RNA was prepared from three independent cultures and each RNA sample was hybridized twice to the microarrays (dye swap). RNA was reverse-transcribed with Superscript indirect cDNA kit (Invitrogen) and labelled with Cy5 or Cy3 (Amersham Biosciences) according to the supplier’s instructions. The microarray contains 6835 60-mer oligonucleotides specific for 2134 predicted genes of the genome of NEM316 strain. The oligonucleotide design was carried out with the OligoArray server [40] and within-house written scripts. The microarray was manufactured by Agilent Technologies. Microarray scanning and results normalization were performed as previously described [41]. To determine differentially expressed genes, we performed a paired t test using the Benjamini and Yekutieli p-value correction method [42] and the cut off for expression ratio was set to either > = 2 or < = 0.5. For each gene, triplicate probes were present on the microarray and the ratio was calculated based on the mean fluorescence intensities of the three probes in each condition. The data of our transcriptome analysis are available in the ArrayExpress database maintained at http://www.ebi.ac.uk/microarray-as/ae/ under the Accession number: E-MTAB-8970

### Construction and complementation of a *rex* null mutant in serotype III strain NEM316 of GBS

For construction of a *rex* deletion mutant (*gbs1167*, GenBank accession no. AL732656), we proceeded as follows: two DNA fragments covering the upstream (Gbs1167-For/Gbs1167intRev) and downstream (Gbs1167intFor/Gbs1167-Rev) regions of the gene were PCR-amplified and then fused by a second PCR with Gbs1167-For and Gbs1167-Rev (S1 Table). The corresponding fragment was cloned into pG+host5 between the EcoRI and BamHI sites [43]. This plasmid was established in *E*. *coli* strain TG1 and then introduced into GBS strain NEM316 by electroporation. Transformants were selected on Ery at 30°C. Plasmid integration in the locus and excision were performed as described [44]. Deletion of *gbs1167* was confirmed by PCR with a primer pair chosen outside the recombination region (gbs1167extFor/gbs1167extRev). For complementation *in trans*, a DNA fragment covering the *rex* gene was PCR amplified with the primer pair Gbs1167cpl For and Gbs1167cpl Rev. This fragment was cloned into pIL252-pBR322 by using the Gibson’s method [45] (S1 Table). The PCR-amplified *re*x gene was cloned into the HindIII site of plasmid pVE3916 (compatible with the pTCV-lac plasmid [46]) using the primers gbs1167HIIIFor and gbs1167HIIIRev (S1 Table). This resulted in plasmid pVE3016-Rex^+^ (pRex).

### Construction of transcriptional fusions and β-galactosidase assays

Promoter regions were PCR amplified with primer pairs for the following genes *gbs0023* (*purC*), *gbs0053* (*adhE*), *gbs0609* (nuclease-like), *gbs1167* (*rex*), *gbs1789* (*menA*), *gbs1388* (*cyl*), *gbs0110*, *gbs1529* and *gbs1929* (*cdnP*) (S1 Table). PCR products were cloned into the EcoRI and BamHI sites of pTCV-lac plasmid [46]. Corresponding plasmids were introduced by electroporation into the WT and its *Δrex* derivative. Cells were grown to the appropriate OD_600_ and 1 ml of culture was collected and centrifuged. Bacterial pellets were frozen for storage. Cells were resuspended in fresh Z buffer and permeabilized by adding ethanol and toluene at 4.5% and 0.5%, respectively [46]. β-galactosidase assays were performed as described in [46] and expressed as specific activity in Miller units (M. U.).

### Expression and purification of native Rex for EMSA, ITC and structural studies

*E*. *coli* TUNER (DE3) cells harbouring the pET101-Strex expression plasmid were grown in 1 L Terrific Broth supplemented with 100 μg.ml-1 ampicillin at 30°C with agitation (200 rpm) until the cultures reached an optical density of 0.8 at 600 nm. Expression of *rex* was induced by addition of 0.4 mM IPTG. The culture was grown for an additional 3 h. Cells were harvested by centrifugation at 8000 *g* for 10 minutes at 4°C. The cell pellets were re-suspended in 50 ml of 50 mM potassium phosphate buffer (pH 7.0) containing 1 mM EDTA. A cocktail of protease inhibitors (Complete, EDTA-free, Roche) was added and the cell suspension was frozen at -20°C. Cells were thawed and lysed by two passages through a French pressure cell operated at 18 000 psi followed by ultra-centrifugation at 100 000 g for 60 min at 4°C. The supernatant containing native Rex was loaded directly onto a Q-Sepharose High Performance anion exchange column (2.6 x 10 cm) pre-equilibrated with 50 mM potassium phosphate buffer (pH 7.0, buffer A). The column was washed with 6 column volumes of buffer A. Rex was eluted with a linear gradient of 0–1 M NaCl in buffer A over 20 column volumes. Fractions were analysed by SDS-PAGE, and those containing Rex eluting between 250–350 mM NaCl were pooled and concentrated. The protein was further purified by gel filtration using a HiLoad (26/600) Superdex 75 pg column. Rex eluted as a single peak using buffer A supplemented with 150 mM NaCl (buffer B). The peak fractions were combined and diluted with an equal volume of 25 mM potassium phosphate buffer (pH 7.0; buffer C) and loaded on to a 5’ AMP-Sepharose 4B column pre-equilibrated with buffer C. Rex was eluted in the apo form using 1 M NaCl in buffer C, in the NAD^+^ bound form using 10 mM NAD^+^ in buffer C, or in the NADH bound form using 10 mM NADH in buffer C. Eluted Rex was concentrated by ultrafiltration (Centriprep concentrators, 10 kDa cutoff, Millipore) to volumes of less than 2 ml. As a final step prior to crystallization, the buffer was exchanged, by repeated concentrations and dilutions, to 50 mM potassium phosphate (pH 7.0) with 100 mM NaCl. The protein was concentrated by ultrafiltration to a final concentration estimated to be 20 mg/ml (BCA assay, Pierce). The protein was stored at +4°C until further use.

### Isothermal titration calorimetry

Isothermal titration calorimetry (ITC) experiments were performed by titrating a nucleotide solution containing either 75 μM NADH, 1 mM NAD^+^, 1 mM NADPH, or 1 mM ATP into a solution with 10 μM freshly isolated apo-Rex. The binding of different DNA fragments to Rex was studied by titrating a Rex solution (100 μM) into a 5 μM DNA-oligonucleotide solution. In the titrations of NAD^+^ into mixtures of Rex and DNA, a solution of 10 μM Rex and 7.5 μM DNA was used in the cell. The titrations were performed in triplicates. All experiments were performed in 100 mM potassium phosphate buffer, pH 7.5 at 25°C using a VP-ITC microcalorimeter (MicroCal, Northampton, MA, USA). The reaction cell contained 1.42 ml of protein in buffer and the reference cell contained distilled H_2_O. After a first injection of 5 μl, 29 injections of 10 μl were made with 5 min spacing between the injections. Data were fitted with the ORIGIN 7 software (MicroCal) using a one-site model. The stoichiometry (*n*) of the binding reaction was set to 1 for the titration with ATP, NAD^+^ and NADPH as well for the titration of NAD^+^ into a mixture of Rex and DNA.

### Crystallization and data collection

Initial crystallization condition screens were made using the crystallization facility at MAX-lab, Lund, Sweden. The PACT premier (Molecular Dimensions, UK) and Hampton Research I and II crystallization screens were used for the initial screening. Drops with a total volume of 600 nl (300 nl protein: 300 nl reservoir solution) were set up in Greiner low profile 96-well plates using a Mosquito robot (TTP LabTech Ltd). Lead conditions were optimized manually using the sitting drop vapor diffusion method at 20°C with crystallization drops containing 1 μl of the protein solution and 1 μl of reservoir solution. *Rex*–*NAD2 crystals*: Rectangular prism-shaped crystals were obtained within two weeks from a drop containing 2 μl of 25 mg/ml protein and 2 μl of reservoir solution containing 180 mM magnesium chloride in 100 mM HEPES buffer at pH 7.0 and 20% (w/v) PEG 6000. *Rex*–*NAD1 crystals*: Cube-shaped crystals of Rex–NAD1 were obtained from 25 mg/ml of Rex mixed with a reservoir solution containing 200 mM sodium fluoride in 100 mM BisTris propane buffer at pH 6.5 and 17% PEG 3350. *Rex*–*DNA complex crystals*: In order to obtain the protein-DNA complex, a self-complementary single stranded oligonucleotide of length 22 bases with the sequence 5’-AATTGTGAAATATTTCACAATT-3’ (purchased from MWG Biotech) was annealed by using a 2 mM solution of single stranded oligonucleotide which was heated to 85°C and allowed to cool down slowly to room temperature over a period of 6 hours. Needle-shaped crystals of the Rex-DNA complex were obtained in a month from a drop containing 2 μl of 1:1 mixture of 29 mg/ml Rex and 1 mM 22-mer DNA duplex and 2 μl of reservoir solution containing 25% PEG 1500 in MIB buffer at pH 5.0. X-ray diffraction data were collected for all the crystals at beamline I911-2 of the MAX-II synchrotron in Lund, Sweden. The data sets were processed using XDS [47]. Crystal data and refinement parameters are given in Table 2.

## Structure solution and refinement

The X-ray structure of Rex–NAD2 was solved by molecular replacement using Phaser [48] interfaced with the CCP4 [49] suite of programs. A model of Rex was derived from the coordinates of *Thermus thermophilus* Rex (PDB entry 2DT5) using Chainsaw [50] and used as the start model for molecular replacement. In the case of the Rex–NAD2 or Rex–DNA complexes, the refined structure of Rex–NAD2 was used as the model for molecular replacement. The asymmetric unit of Rex–NAD1 and Rex–NAD2 consisted of two chains of Rex forming a homodimer, while in the Rex–DNA complex the asymmetric unit consisted of one chain of Rex and a double stranded DNA of 11 base pair length. The solutions obtained from Phaser for the individual structures were improved iteratively by maximum likelihood refinement using Refmac5 [51] and model building by hand using the electron density map generated after a few cycles of refinement and the program Coot [52]. After a few such cycles, the NAD^+^ ligands were located from the difference Fourier map. Rex–NAD2 had two NAD^+^ ligands bound to each of the subunits while Rex–NAD1 had only one. The Rex–DNA complex had only one monomer in the asymmetric unit with one NAD^+^ bound at the nucleotide binding region. The structures were refined to convergence using Refmac5. A total of 552 water molecules, 6 magnesium ions and one chloride ion were located from the electron density map in Rex–NAD2 structure. Fifty-one water molecules were located in the Rex–NAD1 structure and 120 water molecules along with a magnesium ion were located in the Rex–DNA complex structure. Side chain atoms lacking electron density were assigned zero occupancy. The final R-factors were 17.8%, 22.8% and 19.5% with free R-values 24.1%, 29.0% and 24.4% for Rex–NAD2, Rex–NAD1 and Rex–DNA complex structures, respectively. The most important structural and refinement statistics are listed in Table 2.

Rex–NADH model: T-Rex and GBS Rex share a sequence identity of 32.4% and a sequence similarity of 48.4%. A search model for the Rex NADH complex was generated using Chainsaw [50] from the coordinates of the T-Rex–NADH complex (PDB id 2DT5) as template. The non-conserved residues were truncated to the last common atom of their side chains. The structure was solved using molecular replacement, model-built and refined as for the Rex–NAD1 and Rex–NAD2 complexes.

### Electrophoretic mobility shift assays (EMSA)

Protein–DNA interactions were analysed by performing EMSAs. Promoter regions of the potential target genes were amplified by PCR from FTA membranes using specific primers (S1 Table). Amplicons were purified with the Nucleospin Gel and PCR Clean-up kit from Macherey-Nagel according to the manufacturer’s instructions. Purified PCR fragments were incubated with increasing amounts of the GBS Rex protein in reaction buffer containing 20 mM Tris pH 8.0, 1 mM EDTA, 75 mM KCl, 1 mM DTT, 10 μg of Bovine Serum Albumin per ml and 10% glycerol for 20 min at 30°C. When NAD^+^ or/and NADH were included for EMSAs, samples were incubated for an additional 15 min. An internal fragment of 170 bp from the *gbs0455* gene, that does not contain any apparent ROP sequence was used as a DNA competitor (negative control). Reaction mixtures were separated on an 8% polyacrylamide gel in 1X Tris-Borate-EDTA buffer (TBE 1X) for 120 min at 10 mA. After electrophoresis, gels were incubated 15–20 min in TBE 1X containing the GelRed dye (Biotium Inc., Fremont, CA USA) and visualized on a Vilber Lourmat E-Box VX5 imager.

### NAD(H) determinations

Strains were grown in M17rib with 0.5%Glu broth under static condition until OD_600_ of 0.5 in a 5% CO_2_/95% air incubator at 37°C (time T0) and then shifted to aerobiosis (125 rpm on rotary platform) for one hour. At T0 and after one hour of aeration, 1 ml of culture was collected and immediately frozen in liquid nitrogen for storage. Samples were thawed to 4°C, washed with 1 ml of cold water, and pellets were resuspended in 250 μl of 0.2 N HCl or 0.2 N of NaOH for NAD^+^ and NADH extraction, respectively. Once vortexed, samples were boiled at 95°C for 10 minutes. After 10 minutes of centrifugation at 12,000 x g at 4°C, supernatants were transferred into fresh tubes and neutralized with 1 volume 0.2 N of NaOH or HCl for NAD^+^ and NADH solution, respectively. Samples were kept in ice until use. NAD^+^ and NADH determination was performed as described elsewhere [53,54] with some modifications. Briefly, 100 μl of samples were mixed with 100 μl of 1 M Bicine pH 8, 100 μl of 40 mM EDTA (pH 8), 100 μl of 16.6 mM PES (phenazine ethosulfate), 100 μl of pure Ethanol, 100 μl of 4.2 mM MTT (3-(4,5-Dimethyl-2-thiazolyl)-2,5-diphenyl-2H-tetrazolium bromide), and 180 μl of 0.1 M NaCl. After 5 minutes at room temperature, 20 μl of Alcohol dehydrogenase (500 U/ml Sigma A3263) were added and samples were incubated at 37°C in the dark. Concurrently, standard NADH solutions were made to determine NAD^+^ or NADH standard curves amounts in the samples. Reactions were stopped by adding 600 μl of 5 M NaCl and centrifuged at 14,000 x g at 4°C for 10 minutes. Formazan pellets (from MTT reduction) were recovered in 800 μl of pure DMSO. Absorbance was monitored at 570 nm.

### Animal assays

Six-week-old female BALB/c or C57BL/6 mice were purchased from the Animal Resources Centre (Australia). A systemic infection model was used to assess virulence of *S*. *agalactiae*. Bacteria used for infection were grown statically in THB overnight at 37°C, washed twice in PBS (pH 7.4) and resuspended in PBS (pH 7.4) to an optical density of 0.12 at 600 nm. Mice were inoculated through the lateral tail vein with 200 μl of PBS (pH 7.4) containing approximately 6 × 10^6^ bacteria (either the Δ*rex* mutant or the WT strain). Inocula were delivered using 27G × 11/4-in. PrecisionGlide needles (BD) connected to 1-ml tuberculin syringes. At 24 hours post-inoculation, groups of 5–8 mice were sacrificed, and blood was collected by cardiac puncture. Organs were collected, weighed, and homogenized in PBS. The numbers of GBS in blood and organ homogenates were determined by plating on THB agar plates incubated at 37°C. Statistical analysis was performed using the Kruskal-Wallis test (with Dunn’s multiple comparisons), and Mann-Whitney U tests for comparing mutant and WT numbers in separate organs. Animal experiments were repeated twice independently. GraphPad Prism (version 8.0) software was used for all statistical analyses. A P value of <0.05 was considered statistically significant.

## Supporting information

S1 Table Strains, plasmids, and primers. (DOCX)

S2 Table Genes that are up-regulated in the absence of Rex (repression by Rex). (XLSX)

S3 Table Genes that are down-regulated in the absence of Rex (activation by Rex). (XLSX)

S4 Table Genes up-regulated in the absence of Rex. (XLSX)

S5 Table Complementation of *rex* mutant. (DOCX)

S6 Table GBS NEM316 genes with a predicted Rex operator in their promoter region. (DOCX)

S1 Fig Genetic organization of the *rex* locus (*gbs1167*) and similarity to other Rex proteins. A Rho-independent transcription terminator, is present downstream of *gbs1164-1166* operon. Arrow represents the transcriptional start site of the *gbs1167* gene. *gbs1168* encodes a *radC-like* gene. Percentage of identity and protein size (number of amino acid residues) of different Rex proteins are shown.
(EPS)

S2 Fig Role of Rex in gene expression. **A**. Promoter region of Rex controlled genes. Putative Rex binding sites are underlined. −35 and−10 promoter elements are highlighted in grey. Transcription start sites identified by RNA sequencing are indicated by large italic letters. **B.** Activities of the *purC*, *adhE*, *adhP*, *gbs0609*, *rex*, *menA*, *gbs1388*, *gbs0110* and *cdnP* promoter-*lacZ* fusions. Cells were grown in M17 medium supplemented with 0.2% glucose. β-galactosidase activities were determined in log-phase growing cells. Ratios of the activities from the *Δrex* mutant to those of the WT are shown. Experiments were performed three times. Mean values and standard deviations are shown. LacZ activities in the WT strains were: *purC*, ~1 M. U.; *adhE*, ~8 M. U.; *adhP* ~1 M.U. *gbs0609*, ~2 M. U.; *rex* ~1.5 M. U.; *menA*, ~10 M. U.; *gbs1388* ~200 M.U. *gbs0110* ~70 M.U. *cdnP* ~ 30 M. U. For *gbs1529*, no induction was observed (R = 1). P<0.005 was determined by the Kruskal–Wallis nonparametric test, except for *menA*, *gbs1388* and *gbs0110* where P< 0.05.
(EPS)

S3 Fig Deletion of *rex* attenuates virulence in a mouse model. **A**. Complementation of Rex restores wild-type (WT) phenotype in a Δ*rex* mutant. Overnight cultures of the WT, Δ*rex* mutant and complemented mutant (Δ*rex*, *rex*^*c*^) were streaked (left) on sheep blood plate for checking pigment production or (right) on Methyl green agar plate for nuclease activity detection and pigment production. **B.** Effects of Rex on GBS survival in CD1 Swiss mice following intravenous infection. The WT (black) and the Δ*rex* mutant (red) bacteria were grown in THB, collected at the early exponential phase and then washed in PBS buffer before injection of 10^6^ cells per mouse in the bloodstream route in CD1 Swiss mice. The viability was monitored up to 10 days post injection and was represented by Kaplan-Meier’s method using GraphPad Prism (version 8.0) software. Data is the mean of two independent experiments. Statistical analysis was performed using the Chi square test for comparing mutant and WT numbers in mice. **** P value of <0.0001 Chi square method. **C.** Effects of Rex on recovery of GBS from different organs in BALB/c mice following systemic infection; organs were collected 24-hour post-inoculation. Data are from the mean of two independent experiments and show medians with interquartile ranges. *P value of <0.05; **P value of <0.01; ***P value of <0.001; Mann-Whitney U tests.
(EPS)

S4 Fig Isothermal titration calorimetry of Rex with nucleotides and DNA and effect of dithionite of purified Rex. Raw isothermal titration calorimetry data are shown in the top panels. In the lower panels binding isotherms plotted *vs* the molar ratio of titrant fitted using a one-site model are shown. **A**. Sequential injection of a solution with 75 μM NADH into 10 μM Rex (dimer). **B**. Sequential injection of a solution with 1 mM NAD^+^ into 10 μM Rex (dimer). **C**. Sequential injection of a solution with 1 mM NAD^+^ into 5 μM ROP DNA and 10 μM Rex (dimer). **D**. Sequential injection of a solution with 100 μM Rex (dimer) into 5 μM ROP DNA. **E.** Sequential injection of a solution with 1 mM ATP into 10 μM Rex (dimer). **F.** Sequential injection of a solution with 1 mM NADPH into 10 μM Rex (dimer). **G.** Effect of dithionite on purified Rex. Spectrum of 30 μM of Rex in the absence or the presence of dithionite (2 mM). A peak at 340 nm corresponding to the presence of NADH (ε = 6.22 mM cm^-1^) appeared after dithionite reduction. Stoichiometry of Rex/NADH is 1.4.
(EPS)

S5 Fig Electrostatic surface representation of Rex in contact to DNA. Electrostatic surface representation of the structure rotated by 90° relative to Fig 4A from red (negatively charged) to blue (positively charged). In orange, DNA.
(EPS)

S6 Fig Role of Rex in cell physiology. **A.** Deletion of nox2 derepresses *rex* expression. Cells carrying a *rex* promoter-lacZ fusion on plasmid (Prex-lac), were streaked on M17 glucose 0.5% agar plate containing 5-bromo-4-chloro-3-indolyl-β-D-galactopyranoside (X-gal) and incubated 48 hours at 37°C. WT, wild type strain; Δ1, the nox2 mutant. M17 0.5% glucose medium was inoculated with an overnight culture at a cell density of 0.05 (OD_600_), incubated in static condition up to OD_600_ = 0.5 and then shifted to aerobiosis for one hour. Cells carrying the Prex-lac plasmid, were collected just before and one hour after the shift. β-galactosidase (β-gal.) activities are expressed in Miller units (M.U.). Data are the mean of three independent experiments and standard deviations are shown. **B.** Role of *rex* in growth. The WT strain, the Δrex mutant and the complemented mutant (Δrex, rex^c^) were cultured in M17 1% glucose broth under aerated (black circles) and respiratory growth conditions (white circles). **C.** The WT strain (black bar), the Δrex mutant (white bar), and the complemented mutant (Δrex, rex^c^ grey bar) were cultured in M17 1% glucose broth under static (S), aerated (A) and respiratory (R) growth conditions. The optical density (OD600) and pH values were determined from overnight cultures. Results are the mean of three independent experiments and standard deviations are shown. **D.** Growth phenotypes of the WT (black circles), the Δ*rex* mutant (white circles) in TH broth. Results are the mean of three independent experiments and standard deviations are shown. **E.** *rex* expression and catabolite repression by glucose. M17 media supplemented with 0.5% glucose (black bar), 0.5% galactose (white bar) or glucose + galactose (0.25% each, grey bar) were inoculated with WT cells carrying the plasmid Prex-lac. β-galactosidase activities (in Miller units, M.U.) were measured on overnight cultures. Data are means of three independent experiments and standard deviations are shown. **F.** Effect of galactose and glucose on *rex* expression in a Δ*rex* mutant. M17 medium supplemented with 0.5% of glucose (black bars) or galactose (white bars) were inoculated with cells harbouring the Prex-lac plasmid. β-galactosidase activities (M.U.) were determined on cells collected at OD600 = 0.5, early (ES) and late stationary (LS) phases. Mean values and standard deviations of three independent experiments are shown.
(EPS)

S7 Fig Model of Rex regulation by NAD^+^ pool depletion according to carbon sources. **A**. with glucose cells produces significant amount of NAD^+^ to subvert negative effect of NADH on Rex binding; **B**. with galactose, cells do not produce any significant amount of NAD(H), which allows NADH to bind Rex causing derepression of the Rex regulon.
(EPS)
