# Supplementary material for: NAD+ pool depletion as a signal for the Rex regulon involved in Streptococcus agalactiae virulence
Source: PLoS Pathog. 2021 Aug 9;17(8):e1009791. doi: 10.1371/journal.ppat.1009791 (PMC8376106; doi:10.1371/journal.ppat.1009791)
Supplement: S1 Table — (DOCX) [file ppat.1009791.s001.docx]

**S1 Table. Strains, plasmids, primers**

Strains

*Streptococcus agalactiae* (Group B Streptococcus; GBS) NEM316, a capsular serotype III strain with a fully sequenced genome, was used as WT (Glaser et al., 2002)

*E. coli* TG1, F' [*traD36 proAB^+^lacI^q^ lacZ*∆*M15*] *supE thi-1* ∆(*lac-proAB*) ∆(*mcrB-hsdSM*), (r_K-_m_K-_), recipient host used for cloning experiments.

*E. coli* TUNER (DE3) F^-^*ompT* *hsdS*_B_ (r_B_^-^ m_B_^-^) *gal dcm lacY1*(DE3), used for protein production.

Plasmids

pTCVlac Shuttle vector with a *lacZ* reporter gene, Ery^R^ and Kan^R^ (Poyart and Trieu-Cuot, 1997),

pG+host5 derivative of pGK12 plasmid containing the pBR322 replication origin, Ery^R^ (Maguin et al., 1996),

pIL252-pBR322 in *L. lactis* theta replicative plasmid Amp^R^_Ecoli_, Ery^R^_Ecoli, Llactis_ (P. Gaudu)

pVE3916 Cm^R^, rolling circle replicative plasmid (Steen et al., 2005)

pET101 T7 promotor based *E. coli* expression plasmid, Amp^R^ (Thermo Fischer Scientific)

Primers

*rex* mutant

Gbs1167-For GATCGAATTCgaaataaatcttccagactgc

Gbs1167intRev GGTAATGTTAAATGAACTGGGTTAAAAATTTTATTATTCG

Gbs1167intFor cgaataataaaatttttaacccagttcatttaacattacc

Gbs1167-Rev GATCGGATCCGCTAGAACTAGCAAAACGG

gbs1167 extFor TGTATGGAGCCAACTCACCA

gbs1167 ext Rev CCTAGTTGATTTAGGCGCAGA

*rex* complementation

In pBR322-pIL222 as described in NEB protocols (Gibson’s method)

Gbs1167cplFor ATCCGTTAGCGAGGTGCCGCCGGCTAATCTACCGATCATAAAATTC

Gbs1167cplRev AGCTTGGCTGCAGGTCGACGGATCCCTTGTAAAAACATGGCAAC

plasmid For GGATCCGTCGACCTGCAGCCAAGCT

plasmid Rev AGCCGGCGGCACCTCGCTAACGGAT

in pVE3916

gbs1167HIIIFor: ccaagcttaatggacagcctcgttg

gbs1167HIIIRev: ccaagcttctccaaatcccagtgaaag

Overproduction of Rex (Impact method as described NEB protocol)

Txb1-1167FOR GCGCATATGATTATGGATAAGTCTATTCC (Nde1)

Txb1-1167REV3 ATGCAGGAAGAGCCCTGCTGGTTCATGAAATAAAG (Sap1)

*lacZ*-transcriptional fusions in the pTCV*-lac* vector

*purC* (*gbs0023*) 23FOR-Eco 5’-ATGCGAATTCAGAGATTCCAGATGAGGC-3’

23REV-Bam 5’-ATGCGGATCCGCTTGGTCTTTATAGACC-3’

*adhE (gbs0053)* 53FOR-Eco 5’-ATGCGAATTCACAAGTCCGAGTCATGTA-3 ‘

53REV-Bam 5’-GATCGGATCCATCTGTAGTTTCTACC-3’

*adhP* AdhPECOF 5’-ATAGGAATTCATGTTCACCAGCAAATCC-3’

AdhPRBam 5’-TAATGGATCCCTACTTTTACAAGTGCTTC-3’

*gbs0110* ECOFGBS110 5’-ATAGAATTCGATTGAAATACCTAAAGG-3’ BAMRGBS110 5’-ATTGGATCCCCATGTAAGTCAGCATAG-3’

*gbs0609* 609FOR-Eco 5’-ATGCGAATTCTGAGAGTATTTGCATTAG-3’

609REV-Bam 5’-ATGCGGATCCTTCATCTGTTAACCTCT-3’

*gbs0644 (cyl)* CylFOR-Eco 5’-ATGCGAATTCGCTCTTAGATGTGCTTTC-3’

CylREV-Bam 5’-CATTGGATCCTCAAAATATTAGAACGTC-3’

*ldh (gbs0947)* 947FOR-Eco 5’-ATGCGAATTCATTTCACTAGTTAGA-3’

947REV-Bam 5’-TATAGGATCCACCATCACCAACGAGGA-3’

*rex* (*gbs1167*) RexFOR-Eco 5’-ATCGGAATTCGACAGCCTCGTTGAGCG-3’

RexREV-Bam 5’-GATCGGATCCTTTAGGAATAGACTTATC-3’

*gbs1388* 1388EcoFor 5’-ATGGAATTCGTTGGCACTACTTGCTGG-3’

1388BamRev 5’-GATCGGATCCATCATAGAAGCGGTGAG-3’

*gbs1529* 1529ECOF 5’-ATCGGAATTCGGTTTATTAAGCATCAGTTG-3’

1539BAMR 5’-GATCGGATCCTAAATAGATTAAAATGCGAC-3’

*menA* (*gbs1789*) 1789FOR-Eco 5’-GATCGAATTCCAAATTGGAGCTAGTGTTAC-3’

1789REV-Bam 5’-GATCGGATCCCTCTATACATAATCTTCAAC-3’

*cdnP* (*gbs1929*) 1929FOR-Eco 5’- ATGCGAATTCTGACCATAGTTATCCTC-3’

1929REV-Bam 5’- GATTGGATCCGCTGTAGCTGTTAAGACAG-3’

Electrophoretic mobility shift assay

Strepa_Rex1 CACCATGATTATGGATAAGTCTATTCCT

Strepa_Rex2 TTATCGCTGCTGGTTCATGAAATAAAG

Ldh TTTGTTTGCAAATTTTTTCACATTTTAT

Ldh_m1 (C19→T) TTTGTTTGCAAATTTTTTTACATTTTAT

Ldh_m2 (C19→T,C21→T) TTTGTTTGCAAATTTTTTTATATTTTAT

Ldh_m3 (C9→T, A10→G) TTTGTTTGTGAATTTTTTCACATTTTAT

^a^ For oligonucleotides used to construct double stranded probes for EMSA assays only the sequence of the forward oligonucleotide is shown.

references

Glaser, P., Rusniok, C., Buchrieser, C., Chevalier, F., Frangeul, L., Msadek, T., Zouine, M., Couve, E., Lalioui, L., Poyart, C.*, et al.* (2002). Genome sequence of *Streptococcus agalactiae*, a pathogen causing invasive neonatal disease. Mol. Microbiol. *45*, 1499-1513.

Maguin, E., Prevost, H., Ehrlich, S.D., and Gruss, A. (1996). Efficient insertional mutagenesis in lactococci and other gram-positive bacteria. J. Bacteriol. *178*, 931-935.

Poyart, C., and Trieu-Cuot, P. (1997). A broad-host-range mobilizable shuttle vector for the construction of transcriptional fusions to beta-galactosidase in gram-positive bacteria. FEMS Microbiol. Lett. *156*, 193-198.

Steen, A., Buist, G., Horsburgh, G.J., Venema, G., Kuipers, O.P., Foster, S.J., and Kok, J. (2005). AcmA of *Lactococcus lactis* is an N-acetylglucosaminidase with an optimal number of LysM domains for proper functioning. FEBS J. *272*, 2854-2868.
