# Supplementary material for: NAD+ pool depletion as a signal for the Rex regulon involved in Streptococcus agalactiae virulence
Source: PLoS Pathog. 2021 Aug 9;17(8):e1009791. doi: 10.1371/journal.ppat.1009791 (PMC8376106; doi:10.1371/journal.ppat.1009791)
Supplement: S5 Table — (DOCX) [file ppat.1009791.s005.docx]

**S5 Table. Rex complementation**

**__________________________________________________________________________________**

Promoter fusion *Δrex* + pVE3016 *Δrex* + pRex WT

*adhE* 3200 ± 160 190 ± 20 18 ± 2

*cdnP* 1700 ± 200 150 ± 30 26 ± 3

GBS *Δrex* cells harboring the promoter-*lacZ* fusions carried on the pTCV vector were transformed with plasmid pVE3016 or pVE3016-Rex^+^ (pRex). β-galactosidase activity was assessed in late exponential growth phase. Wild-type (WT) cells transformed with the pTCV vector carrying the promoter-*lacZ* fusions were used as a control. Data are the mean of three experiments and standard deviations are shown.
