# Supplementary material for: NAD+ pool depletion as a signal for the Rex regulon involved in Streptococcus agalactiae virulence
Source: PLoS Pathog. 2021 Aug 9;17(8):e1009791. doi: 10.1371/journal.ppat.1009791 (PMC8376106; doi:10.1371/journal.ppat.1009791)
Supplement: S6 Table — (DOCX) [file ppat.1009791.s006.docx]

**S6 Table. GBS NEM316 genes with a predicted Rex operator in their promoter region**

---------------------------------------------------------------------------------------------------------------------------

Gene Encoded function

---------------------------------------------------------------------------------------------------------------------------

*gbs0023** PurC, phosphoribosylaminoimidazole-succinocarboxamidesynthase

*gbs0042** PurD, phosphoribosylamine--glycine ligase

*gbs0053* AdhE aldehyde-alcohol dehydrogenase

*gbs0054* AdhP, alcohol dehydrogenase (propanol-preferring)

*gbs0268* transketolase [EC:2.2.1.1]

*gbs0553* pyrD, dihydroorotate dehydrogenase A

*gbs0583* adenosine deaminase [EC:3.5.4.4]

*gbs0608* enolase

*gbs0609* putative nuclease

*gbs0644** CylX, biosynthesis of the Cyl beta-hemolysin/cytolysin

*gbs0789* oxalate/formate antiporter

*gbs0895** pyruvate dehydrogenase E1 component alpha subunit

*gbs0947* Ldh, L-lactate dehydrogenase

*gbs1082** PyrF, orotidine-5'-phosphate decarboxylase

*gbs1167* Rex , transcriptional repressor

*gbs1174* Voltage-gated chloride channel family protein

*gbs1388** hypothetical protein (in operon with a haemolysin III protein)

*gbs1403* NudP, 5’ -nucleotidase

*gbs1422* phosphotransferase system, EIIC family protein

*gbs1462** Fe complex transport system ATP-binding protein

*gbs1809* Pgk, phosphoglycerate kinase

*gbs1929* CdnP, 2 ,3 -cyclic-nucleotide 2 -phosphodiesterase

* first gene of an operon
